# Chylous Ascites: A Rare Adverse Effect of Methimazole Treatment for Grave's Disease—A Case Report and Review of the Literature

**DOI:** 10.1155/2015/649303

**Published:** 2015-08-23

**Authors:** Tawfik Khoury, Ronen Schneider

**Affiliations:** ^1^Department of Medicine, Hadassah-Hebrew University Medical Center, P.O. Box 12000, IL-91120 Jerusalem, Israel; ^2^Department of Nephrology, Hadassah-Hebrew University Medical Center, P.O. Box 12000, IL-91120 Jerusalem, Israel

## Abstract

A 40-year-old woman was admitted due to an urticarial rash that was attributed to recent onset of methimazole treatment for a diagnosis of Grave's disease. The patient had no prior significant medical history and used no medications, including over-the-counter or herbal medications. Her sister had Grave's disease. On admission, the patient received corticosteroids with improvement in her rash. On the second day of the hospitalization, the patient complained of abdominal discomfort. Abdominal ultrasound revealed a large amount of new onset ascites. Peritoneal tap yielded a milky fluid with high triglyceride level (12.2 mmol/L or 1080 mg/dL), consistent with chylous ascites. After discontinuation of the methimazole, the ascites disappeared. The patient later underwent therapeutic thyroidectomy, after which all features of thyrotoxicosis had improved.

## 1. Introduction

Hyperthyroidism can be a result of several disease states including Grave's disease, toxic adenoma, toxic multinodular goiter, Hashimoto's thyroiditis, and more. Graves' disease is the most common cause (95%) of hyperthyroidism, an autoimmune disorder resulting from thyrotropin receptor stimulation by autoantibodies [1]. Some of the manifestations of Grave's disease, like warm skin and sweating, stare and lid lag, increased cardiac output, tachycardia, and congestive heart failure are common to the other etiologies, but some are unique. Among the unique features are ophthalmopathy and infiltrative dermopathy [2]. In addition, rare manifestations of Grave's disease have been described, including pulmonary hypertension and chylous ascites, for which we found only one case reported in the literature [3].

Treatment of Grave's disease may consist of antithyroid drugs such as methimazole and propylthiouracil (PTU) which are thionamide drugs. Among the adverse events of antithyroid treatment are fever, rash, agranulocytosis, and hepatitis. These reactions usually occur within the first few months on initiating treatment [4]. We could not find literature relating chylous ascites to methimazole or PTU treatment.

In this report we describe the first case of chylous ascites secondary to antithyroid treatment with methimazole for hyperthyroidism with atrial fibrillation and pulmonary hypertension.

## 2. Case Presentation

A previously healthy 40-year-old woman presented with one-week history of diffuse urticarial rash to the dermatology ward which was successfully treated with systemic steroids and antihistamine medications. Three weeks prior to admission, treatment with methimazole 10 mg twice daily had been initiated due to a presumptive diagnosis of Grave's thyroiditis. The diagnosis was made in an outpatient clinic according to typical clinical picture, with low TSH level 0.01 MU/L (normal range 0.35–5.5 MU/L) and high T4 level of 59.1 Pmol/L (normal range 10–20 Pmol/L).

On admission the patient had no symptoms suggestive of hyperthyroidism; on physical examination, an extensive urticarial rash was observed on the limbs and trunk. The patient had a blood pressure of 131/60 mm/Hg, temperature of 36.°C, respiration rate of 16 breaths/min, and a pulse rate of 83 beats/min. Her hands were warm and displayed resting tremor. Cardiac examination was normal, but jugular venous distention was noted. Lungs were clear to auscultation. There was no peripheral edema. Extra ocular movements were normal. There was no proptosis or periorbital edema. Thyroid gland was not tender, was slightly enlarged, and had a rubbery consistency on palpation.

Laboratory examinations revealed a white blood cell count: 15.5 × 10^9^ cells/millilitre (normal range: 4–10 × 10^9^ cells/millilitre) with 90% neutrophils and hemoglobin was 12.7 g/dL. On the second day of her hospitalization the patient began to complain of worsening abdominal pain. Liver function tests were taken initially on day two and showed mild abnormality in alanine aminotransferase (ALT), aspartate aminotransferase (AST), and gama-glutamil transferase (GGT) levels (86, 72, and 88 U/L, resp.). At this time point, methimazole treatment was discontinued. On the third day of the hospitalization enzyme levels were as follows: ALT 270 U/L, AST 430 U/L, and GGT 210 U/L (normal ranges 0–40 U/L for ALT, 0–35 U/L for AST, and 5–36 U/L for GGT). T4 level was 47 Pmol/L (normal range 10–20 Pmol/L) and T3 level was 5.5 Nmol/L (normal range 1.2–3 Nmol/L).

An abdominal ultrasound showed a large amount of ascites. A diagnostic tap showed fluid with a milky white appearance. Triglyceride level was 12.2 mmol/L (1080 mg/dL), and total protein and lactate dehydrogenase (LDH) levels in ascites were 3.3 g/dL and 326 U/L, respectively. Gram stain was negative for bacteria and fluid cultures were negative. Purified protein derivative (PPD) skin test, acid fast stains of peritoneal fluid, and fluid cultures for tuberculosis were all negative. Serologies for hepatitis A virus, hepatitis B virus, and hepatitis C virus were all negative. Serologies for Epstein-Barr virus and Cytomegalovirus were consistent with prior exposure. Abdominal computed tomography (CT) showed peritoneal fluid, an undefined ovarian mass on the right, and no other abnormalities. Liver size and consistency were normal. Vaginal US did not define the mass better. A lymphangiogram showed no anatomical abnormalities of abdominal lymph vessels or nodes. A gynecological exam was normal, including vaginal ultrasound (US) to define the ovarian mass found on CT scan. The ovarian mass represented a corpus luteum most probably. A follow-up abdominal US after four days showed that the peritoneal fluid had disappeared.

In parallel to this workup the patient started developing signs and symptoms of thyrotoxicosis such as rapid atrial fibrillation which was not present before the discontinuation of methimazole, worsening tremor, and moderate pulmonary hypertension diagnosed with echocardiography. Since treatment with other antithyroid drugs was considered dangerous and since treatment with iodine was expected to have influence only after 4–8 weeks, and the patient had severe symptoms, a therapeutic thyroidectomy was performed two months after admission.

After the procedure the patient was free of symptoms and a follow-up echocardiography showed marked improvement in her pulmonary hypertension. After 1 year of follow-up the patient was asymptomatic, without pulmonary hypertension and with no peritoneal fluid.

## 3. Discussion

We report a case of Grave's disease treated with methimazole with two rare and interesting features. The first is chylous ascites which is an uncommon finding.

It is usually caused by a chronic disruption of the lymphatic system. Several mechanisms had been proposed for the pathophysiology of chylous ascites, including exudation of lymph material through the walls of acquired or congenital dilated retroperitoneal vessels into the abdominal cavity, direct trauma of the thoracic duct, and obstruction of the lymph flow through the dilated subserosal lymphatics into the peritoneal cavity which produces collagen deposition, fibrosis, and protein-losing enteropathy [5]. The diagnosis is based on the biochemical study of the ascetic fluid. The most important diagnostic criterion is the presence of >2.3 mmol (>200 mg/dL) of triglycerides in ascites fluid [6–9].

There are several causes of chylous ascites; among them are many pathological processes such as infections, cirrhosis, malignancy, congenital defects, inflammatory process, trauma, and cardiac and renal diseases. Almost two-thirds of all chylous ascites present in developed countries as a consequence of abdominal malignancy [6, 10] and cirrhosis [11–14]. On the other hand, infectious etiologies, such as tuberculosis or filariasis, are responsible for the majority of the cases in developing countries [15, 16].

The cornerstone management in chylous ascites is treating the underlying cause whenever possible [17–19]. The resolution of chylous ascites usually takes several weeks [20, 21]. But in our case, it resolved few days after the discontinuation of methimazole. Furthermore, when ascites persists after the resolution of the underlying cause, it is recommended to provide a low lipid diet and high protein diet: the former should be supplied in the form of medium chain triglyceride (MCT) which is directly absorbed to the portal circulation from the intestine bypassing the lymph nodes and it reduces the production and flow of lymph. On the other hand, long chain triglycerides in diet must be avoided, as these are converted into free fatty acids and monoglycerides which are transported through the lymphatic system [17, 18, 20]. Moreover, somatostatin analogs which have an inhibitory effect on gastrointestinal hormones and processes have been successfully used in the treatment of chylous ascites, although the mechanism of action in these disorders remains unclear and needs further clarification [22–25]. And lastly, patients who do not improve with enteral nutrition and somatostatin analogs can be managed with total parenteral nutrition with variable results [26–28].

The etiologic list guided our workup as described above. Although the patient was an Israeli resident, she had emigrated from the Philippines two years prior to admission, and hence an infectious workup was carried out. As mentioned, no evidence of malignancy or intra-abdominal infection was found, and no evidence of anatomical aberrations in lymph structures, including lymphangioleiomyomatosis, was found, using imaging modalities such as abdominal ultrasound, CT scan, and lymphangiogram. The negative workup, and the disappearance of ascites in parallel to improvement in LFTs after discontinuation of methimazole, led us to the conclusion that hepatitis and chylous ascites in our patient were secondary to methimazole treatment.

After extensive medical database search on Medline and Embase we could not find such a relation with chylous ascites described in the literature. Thus, our case is the first case describing the causative association between methimazole and chylous ascites.

The second interesting phenomenon presented by our patient was pulmonary hypertension secondary to thyrotoxicosis. This is much better described in the literature but still represents an uncommon finding [29]. Pulmonary hypertension in our patient resolved completely after thyroidectomy, emphasizing the relation of these findings.

In conclusion, this case emphasizes the diversity of possible adverse events of antithyroid drugs and the diverse presentation of Grave's disease.
